# Occallatibacter bavaricus sp. nov., a new representative of the Acidobacteriota isolated from fen soils, reclassification of Terracidiphilus gabretensis as Occallatibacter gabretensis comb. nov. and emended description of the genus Occallatibacter

**DOI:** 10.1099/ijsem.0.007086

**Published:** 2026-02-25

**Authors:** Katharina J. Huber, János Papendorf, Carolin Pilke, Petra Büsing, Boyke Bunk, Cathrin Spröer, Sarah Kirstein, Jacqueline Wolf, Meina Neumann-Schaal, Manfred Rohde, Michael Pester

**Affiliations:** 1 Department of Microorganisms, Leibniz Institute DSMZ – German Collection of Microorganisms and Cell Cultures, Braunschweig, Germany; 2 Bioinformatic Services, Leibniz Institute DSMZ – German Collection of Microorganisms and Cell Cultures, Braunschweig, Germany; 3 Department of Metabolomics & Services, Leibniz Institute DSMZ – German Collection of Microorganisms and Cell Cultures, Braunschweig, Germany; 4 Braunschweig Integrated Centre of Systems Biology (BRICS), Braunschweig, Germany; 5 Department of Medical Microbiology, Central Facility for Microscopy, Helmholtz Centre for Infection Research, Braunschweig, Germany; 6 Chair of Microbial Physiology, Technical University of Munich, Freising, Germany

**Keywords:** *Acidobacteriota*, fen soils, soil bacteria

## Abstract

The acidobacterial strain JP12^T^ affiliated with the *Terriglobales* has been isolated from fen soil sampled at Schlöppnerbrunnen II near Bayreuth, Germany. The strain was Gram-stain-negative, non-motile, displayed non-spore-forming rods that divide by binary fission and segregate exopolysaccharide-like structures. JP12^T^ grew at temperatures of 4–40 °C (best between 24 and 30 °C), at pH values of 3.7–6.0 (best between 4.1 and 5.6) and at NaCl concentrations of 0–0.5% (best 0.25%, w/v).

MK-8 was identified as the major respiratory quinone. The major fatty acids of the strain JP12^T^ were *iso*-C_15:0_, C_16:0_, *iso*-C_17:1_ *ω*7*c*, *iso*-C_17:0_ and *iso*-diabolic acid. Phosphatidylglycerol, phosphatidylethanolamine and ornithine-containing lipids were the major polar lipids. Lysophosphatidylethanolamine, phosphatidylinositol, diphosphatidylglycerol and high-mass intact polar lipids occurred in small amounts. The G+C content of the strain JP12^T^ was 56.4 mol%. 16S rRNA gene sequence similarity values of 96.1–96.7% placed the strain JP12^T^ in the vicinity of the type strains of *Occallatibacter savannae* A2-1c^T^, *Telmatobacter bradus* TPB6017^T^, *Occallatibacter riparius* 277^T^ and *Terracidiphilus gabretensis* S55^T^, respectively. Based on the phenotypic, phylogenetic and chemotaxonomic data, we propose the new species *Occallatibacter bavaricus* sp. nov. (type strain JP12^T^=DSM 110680^T^=CECT 30267^T^) within the acidobacterial order *Terriglobales*. We also propose the reclassification of *Terracidiphilus gabretensis* as *Occallatibacter gabretensis* comb. nov.

## Data Summary

All supporting data have been provided within the article or through supplementary data files.

## Introduction

Although the *Acidobacteriota* represent one of the most abundant fractions of the active bacterial community in different soil environments [1,3], comparably low numbers of described strains are available. Due to improved cultivation attempts over the past years, the number of described species has been increased to 81. A high percentage of the known and described acidobacterial species belongs to the genera *Granulicella* [4], *Telmatobacter* [5], *Edaphobacter* [6], *Tunturiibacter* [7] and *Terriglobus* [8] within the order *Terriglobales* [9]. While the genera *Edaphobacter*, *Tunturiibacter*, *Granulicella* and *Terriglobus* harbour at least four species, only one or two species are described for the genera *Occallatibacter* [10]*, Telmatobacter* [5] and *Terracidiphilus* [11] so far. Interestingly, *Terriglobales* dominate in soils with low pH (~3.7–5.0), such as fen and peatland soils [12,15], and display different metabolic potentials, such as polysaccharide hydrolysis, sugar utilization, aerobic respiration, several fermentative capabilities and hydrogen oxidation [15,17], which were proven by physiological characteristics of isolated species or indicated by genomic analyses.

*Terriglobales* strains were mainly isolated by the use of specialized media imitating nutrient content, pH value, oxygen relation and temperature of the original habitat. However, soil bacteria are subjected to varying environmental conditions, such as water availability, temperature changes and oxygen concentrations caused by seasonal changes and weather impacts. Fen soils, which are characterized by low and stable pH values, high carbon content and high water saturation, are also directly influenced by seasonal changes and precipitation values. Therefore, varying conditions during isolation experiments, i.e. oxygen availability, would better imitate soil conditions and could enable the isolation of previously unculturable acidobacterial strains.

In the present study, we describe one new representative of the *Acidobacteriota* isolated from fen soil, representing a new species within the genus *Occallatibacter*, within the order *Terriglobales*, isolated by the use of varying oxygen conditions during the cultivation process.

## Origin and isolation

Strain JP12^T^ was isolated from a soil sample that was collected from the Schlöppnerbrunnen II fen (50° 8′ 8.27″ N 11° 52′ 48.3″ E) close to Bayreuth, Germany, in December 2018, and was subsequently stored at 4 °C. In the laboratory, 5 g of fen soil (pH 4–5) was mixed with 49.5 ml of MES buffer (10 mM, pH 5.0), serially diluted and inoculated in SSE/HD 1:10 medium (DSMZ medium 1426 – https://www.dsmz.de/microorganisms/medium/pdf/DSMZ_Medium1426.pdf; Leibniz-Institute DSMZ - German Collection of Microorganisms and Cell Cultures GmbH). The SSE/HD 1:10 medium contained soil solution equivalent (SSE) [18], was supplemented with a 10-vitamin solution [19] and a trace element solution SL-10 [20], and was solidified with purified agar (Oxoid, Basingstoke, UK), if indicated. 180 µl SSE/HD 1:10 medium (pH 5.0) were mixed with 20 µl of the respective soil solution suspensions in 96-well plates and incubated at 20 °C in the dark under changing oxygen conditions – 1 week under oxic conditions (normal laboratory conditions; 21% O_2_) and 1 week under a microaerobic atmosphere (candle jar technique; 3–5% O_2_) – without shaking. Additionally, 100 µl of the serial dilution step 10^-3^ were equally spread on SSE/HD 1:10 medium plates (pH 5.0) and incubated as described above. After 4 weeks of incubation, 26 turbid wells of the liquid-medium approach and 42 colonies of the solid-medium approach were screened for the presence of *Acidobacteriota* by PCR employing the primer pair 27f and 1492r [21] (see Supplementary Material for details) and subsequent Sanger sequencing. Thirteen *Acidobacteriota*-positive cultures were purified by subsequent restreaking on SSE/HD 1:10 agar plates until strain JP12^T^ was isolated, which was checked by colony PCR and Sanger sequencing.

## 16S rRNA gene phylogeny

The 16S rRNA gene sequence of JP12^T^ was amplified by colony PCR employing the primer pair 8f [22] and 1492r [21] and subsequent Sanger sequencing. blast (National Center for Biotechnology Information) analysis placed JP12^T^ in the vicinity of the type strains of *Occallatibacter savannae* A2-1c^T^ (16S rRNA sequence identity value of 96.7%), *Telmatobacter bradus* TPB6017^T^ (96.4%), *Occallatibacter riparius* 277^T^ (96.3%) and *Terracidiphilus gabretensis* S55^T^ (96.1%).

**Fig. 1.**
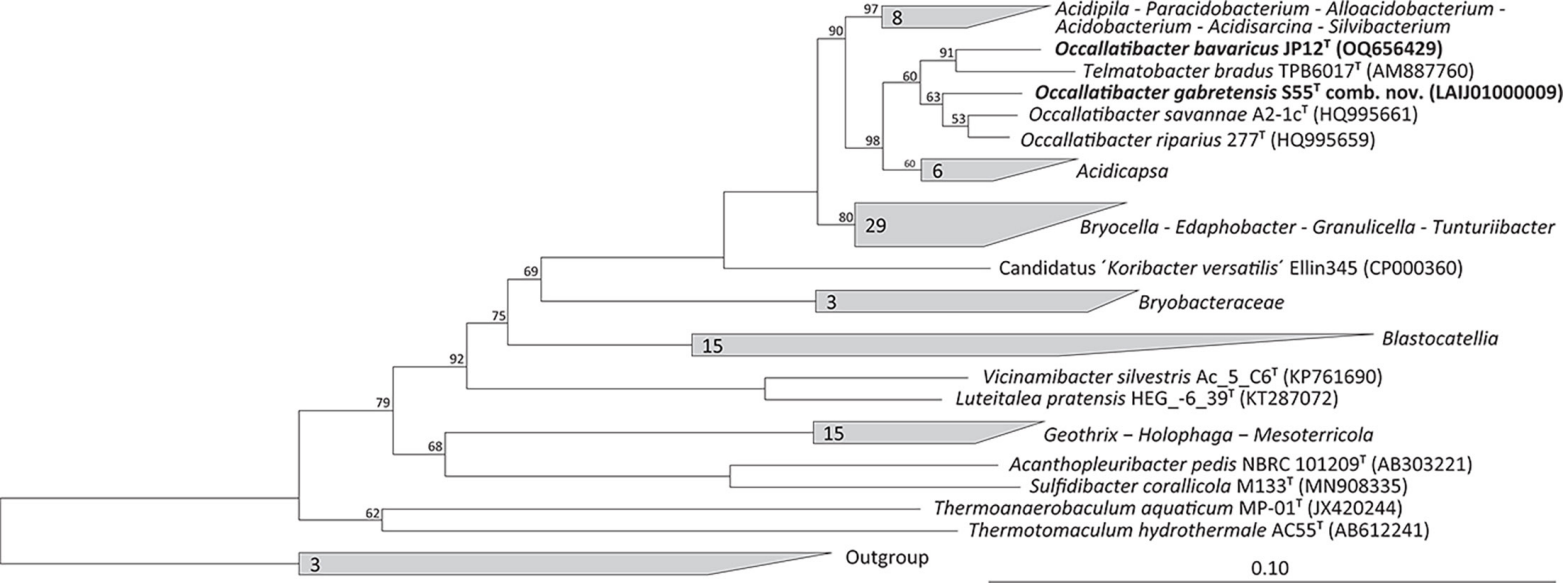
Rooted neighbour-joining phylogenetic tree (Felsenstein correction) based on almost full-length 16S rRNA gene sequences showing the relationship of strain JP12^T^ and related type strains. Bootstrap values are expressed as percentages of 1,000 replicates and are indicated at the respective branching points. The following sequences were used as outgroups: *Novipirellula rosea* LHWP3^T^ (JF748734), *Blastopirellula marina* DSM 3645^T^ (X62912) and *Pirellula staleyi* DSM 6068^T^ (CP001848). The bar indicates 10% nucleotide divergence.

The 16S rRNA gene sequence of JP12^T^ was added to the small subunit ribosomal RNA non-redundant reference database silva version 138.1 (https://www.arb-silva.de/) via the programme package arb for phylogenetic tree calculations. The alignment was corrected manually based on the secondary-structure information after the implementation by the automated alignment. All three different algorithms, neighbour-joining, maximum-parsimony and maximum-likelihood algorithms (termini filter; 1,376 valid columns between position 63 and 1,439 of the *Escherichia coli* 16S rRNA reference gene; 1,000 bootstrap resamplings), placed JP12^T^ within the *Terriglobales*, in the vicinity of the strains *T. bradus* TPB6017^T^, *O. savannae* A2-1c^T^, *O. riparius* 277^T^ and *T. gabretensis* S55^T^ (Figs 1, S1 and S2, available in the online Supplementary Material).

## Ecological distribution

To examine the distribution of strain JP12^T^ in the environment, the 16S rRNA gene sequence of JP12^T^ was compared via blast (https://blast.ncbi.nlm.nih.gov/Blast.cgi) against acidobacterial sequences from cultured and uncultured environmental samples. Interestingly, all 16S rRNA gene sequence hits with identity values above 99.0% derived from soil samples from German forest soils [23], polychlorinated biphenyl-polluted soil [24], oak forest soil [25], pristine soil [26] and Australian pasture soils [27], indicating an adaptation of JP12^T^ and closely related sequence types to the soil environment.

## Phylogenomic placement

The DSMZ sequencing service department sequenced the genome of JP12^T^ on the PacBio Sequel IIe instrument (Pacific Biosciences, Menlo Park, CA, USA; details of the methods used are given in the Supplementary Material). The raw sequence data were quality checked, filtered and resulted in 80,563 sequence reads with a mean subread length of 4,324 bp. The HGAP version 4 protocol included in the SMRT Link analysis software assembled the genome of JP12^T^, which finally displayed a length of 5,987,865 bp and a G+C content of 56.4 mol%. As high sequencing coverage values could be obtained (487 mean coverage), a correction of the genome by short-read sequencing was not necessary. After the assembly, the genome was automatically oriented after *oriC* and subsequently analysed with the Prokka pipeline, resulting in 5,040 coding sequence regions, 3 rRNAs and 76 tRNAs (Table S1). CheckM analysis (v1.2.2) provided by NCBI (National Center for Biotechnology Information) confirmed 97.22% completeness and 2.4% contamination of the genome of JP12^T^.

**Fig. 2.**
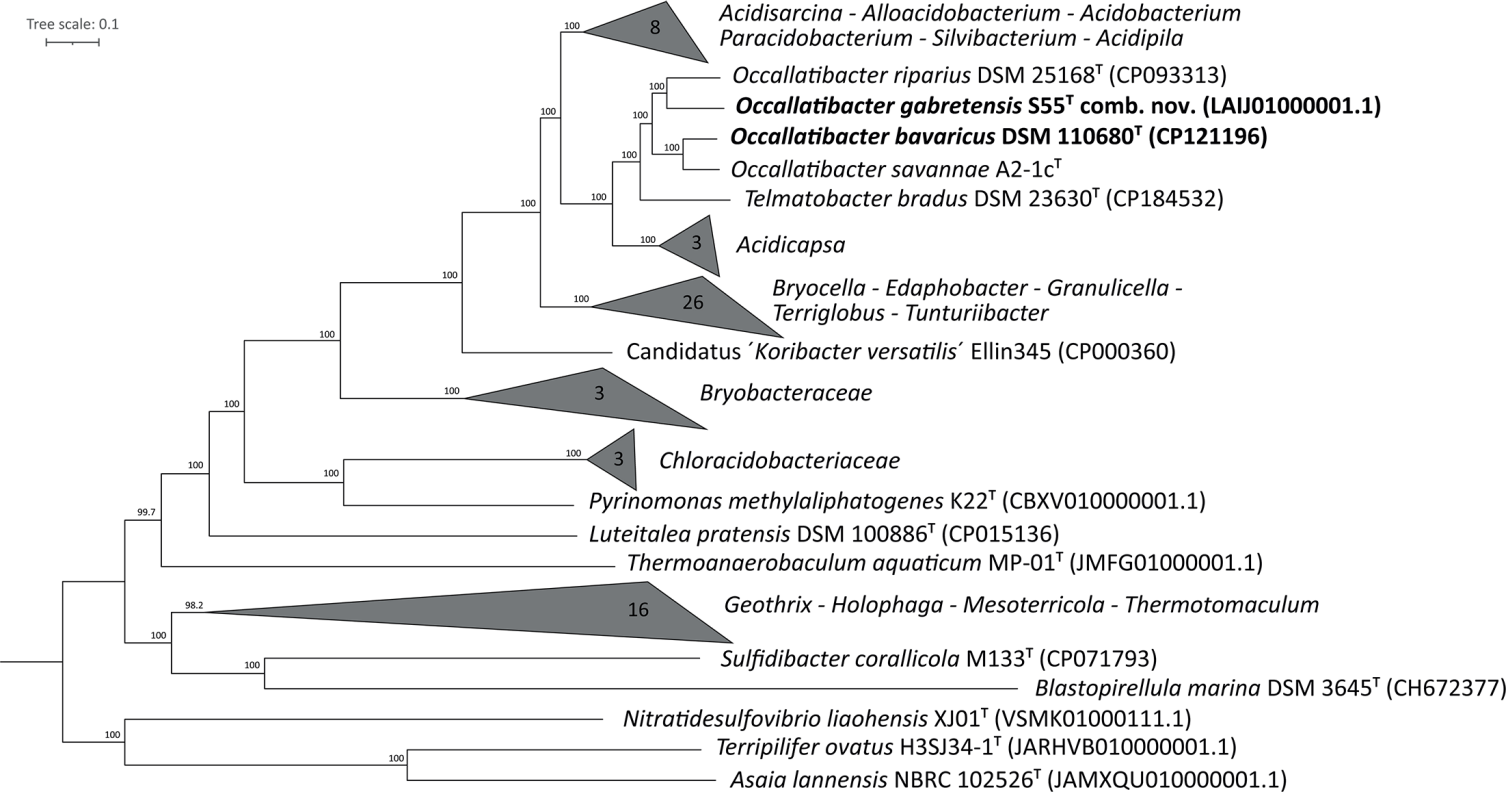
Phylogenomic reconstruction of the *Acidobacteriota* based on a concatenated alignment of 120 single-copy bacterial marker proteins. *Blastopirellula marina* DSM 3645^T^, *Nitratidesulfovibrio liaohensis* XJ01^T^, *Terripilifer ovatus* H3SJ34-1^T^ and *Asaia lannensis* NBRC 102526^T^ were used as outgroups. The bar indicates 10% sequence divergence.

To confirm the phylogenetically closest relatives of JP12^T^, a phylogenomic tree was calculated based on 120 single-copy marker genes of all available acidobacterial type strain genomes, which were extracted and aligned with GTDB-Tk v2.4.0 [28]. The tree calculation was performed with IQ-TREE 2, multicore version 2.4.0, using ultrafast bootstraps (*n*=1,000) after automatic substitution model selection, selecting LG+F+R9 as the best-fit model [29]. The resulting maximum-likelihood tree identified *Occallatibacter savannae* A2-1c^T^, *O. riparius* DSM 25168^T^ and *Terracidiphilus gabretensis* S55^T^ as the phylogenetically closest relatives of JP12^T^ (Fig. 2).

## Whole-genome similarity indices

Genome similarity indices of JP12^T^ and its phylogenetically closest relatives were used for species and genus demarcation. Average nucleotide identity (ANI), average amino acid identity (AAI), digital DNA–DNA hybridization (dDDH) and percentage of conserved proteins (POCPs) were calculated employing the OrthoANIu algorithm via the EzBioCloud web service webpage (https://www.ezbiocloud.net/tools/ani) [30], the EzAAI tool [31], the Genome-to-Genome Distance Calculator version 3.0 (https://ggdc.dsmz.de/ggdc.php) and the POCP-nf pipeline (https://github.com/hoelzer/pocp?tab=readme-ov-file#run) [32,35], respectively.

ANI values of 71.8%, 73.9%, 71.2% and 71.2%, as well as dDDH values of 20.7%, 19.5%, 19.3% and 20.6%, of strain JP12^T^ compared with *Occallatibacter riparius* 277^T^ and 307, *O. savannae* A2-1c^T^, *Terracidiphilus gabretensis* S55^T^ and *Telmatobacter bradus* DSM 23630^T^ (Table 1) clearly showed that JP12^T^ represents a new species within the *Telmatobacter*–*Occallatibacter*–*Terracidiphilus* clade.

**Table 1.** Overview of the whole-genome similarity indices of strain JP12^T^ compared with its phylogenetically most closely related type strains Strains: 1, JP12^T^; 2, *Occallatibacter riparius* DSM 25168^T^/307 [10]; 3, *O. savannae* A2-1c^T^ [10]; 4, *Terracidiphilus gabretensis* S55^T^ [11] and 5, *Telmatobacter bradus* DSM 23630^T^ [5]. The values are given in percentages and were obtained during the current study. Values of JP12^T^ in comparion to other strains are highlighted in bold.

Characteristic	1	2	3	4	5
	**16S rRNA**				
**1**	**100**	**96.28**	**96.73**	**96.13**	**96.42**
**2**	**96.28**	100	98.29	97.71	96.09
**3**	**96.73**	98.29	100	97.00	95.31
**4**	**96.13**	97.71	97.00	100	95.27
**5**	**96.42**	96.09	95.31	95.27	100
	**ANI**				
**1**	**100**	**71.82/71.76**	**73.94**	**71.16**	**71.17**
**2**	**71.82/71.76**	100/97.45	72.71/72.85	72.68/72.72	71.68/71.85
**3**	**73.94**	72.71/72.85	100	72.13	71.11
**4**	**71.16**	72.68/72.72	72.13	100	71.29
**5**	**71.17**	71.68/71.85	71.11	71.29	100
	**dDDH**				
**1**	**100**	**20.70/20.30**	**19.50**	**19.30**	**20.60**
**2**	**20.70/20.30**	100/93.00	13.40/13.40	13.30/13.20	13.10/13.00
**3**	**19.50**	13.40/13.40	100	13.00	12.90
**4**	**19.30**	13.30/13.20	13.00	100	12.90
**5**	**20.60**	13.10/13.00	12.90	12.90	100
	**AAI**				
**1**	**100**	**70.01/70.00**	**76.79**	**69.22**	**68.08**
**2**	**70.01/70.00**	100/98.33	69.80/69.76	69.88/69.82	67.54/67.56
**3**	**76.79**	69.80/69.76	100	69.03	67.77
**4**	**69.22**	69.88/69.82	69.03	100	67.24
**5**	**68.08**	67.54/67.56	67.77	67.24	100
	**POCP**				
**1**	**100**	**60.98/60.88**	**69.61**	**56.33**	**45.90**
**2**	**60.98/60.88**	100/92.47	63.99/63.64	56.34/56.11	45.42/44.25
**3**	**69.61**	63.99/63.64	100	54.12	45.25
**4**	**56.33**	56.34/56.11	54.12	100	48.44
**5**	**45.90**	45.42/44.25	45.25	48.44	100

AAI values of 70.0% and 76.8% for strain JP12^T^ compared with *O. riparius* 277^T^ and *O. savannae* A2-1c^T^, respectively, exceeded the suggested genus demarcation threshold of 68% [36,37]. In addition, an AAI value of 69.22% between JP12^T^ and *T. gabretensis* S55^T^ slightly exceeded the genus demarcation threshold. Interestingly, an AAI value of 69.9% between *O. riparius* 277^T^ and *T. gabretensis* S55^T^ and an AAI value of 69.0% between *O. savannae* A2-1c^T^ and *T. gabretensis* S55^T^ would suggest that *T. gabretensis* S55^T^ also belongs to the genus *Occallatibacter*. Therefore, strain JP12^T^ represents a new species within the *Occallatibacter* genus.

An AAI value of 67.5% between *O. riparius* 277^T^ and *T. bradus* DSM 23630^T^ and an AAI value of 67.8% between *O. savannae* A2-1c^T^ and *T. bradus* DSM 23630^T^ fell slightly below the suggested genus demarcation value of 68%, confirming that *T. bradus* DSM 23630^T^ represents a separate genus within the *Occallatibacter*–*Telmatobacter*–*Terracidiphilus* clade, whereas an AAI value of 68.08% between JP12^T^ and *T. bradus* DSM 23630^T^ slightly exceeded the suggested threshold of 68% for genus demarcation (Table 1).

To support the findings of the AAI values, POCP values were additionally calculated. Strain JP12^T^, the three *Occallatibacter* strains and *T. gabretensis* S55^T^ displayed POCP values of 54.12–92.47% among each other, while *T. bradus* DSM 23630^T^ showed POCP values of 45.25–48.44% compared with JP12^T^, the three *Occallatibacter* strains and *T. gabretensis* S55^T^ (Table 1). The suggested POCP demarcation value for the definition of a genus is 50% [35]. Therefore, the calculated POCP values confirmed that strains JP12^T^ and *T. gabretensis* S55^T^ would represent species of the *Occallatibacter* genus and that *T. bradus* DSM 23630^T^ would further represent a separate genus within the *Terriglobales*.

## Genomic features

The annotation and analysis platform implemented on the Bacterial and Viral Bioinformatics Resource Center (BV-BRC) webpage assigned 22% of the genome sequence of JP12^T^ to known subsystem categories. The groups with the most assigned genes were ‘protein synthesis’ (113 genes), ‘cofactors, vitamins, prosthetic groups’ (81), ‘amino acids and derivatives’ (111) and ‘stress response, defence and virulence’ (94). While most of the known and strongly represented subsystem categories contained genes for the ‘normal’ metabolism, higher gene numbers of ‘stress response, defence and virulence’ would explain the broad temperature and pH growth and tolerance ranges detected for JP12^T^ (see Section Growth ranges). As fen soils, such as Schlöppnerbrunnen II, from which JP12^T^ was isolated, are characterized by high carbon content, high water saturation and underlie seasonal fluctuations in temperature and water availability, strain JP12^T^ had to be adapted to changing temperature values and also pH values because of changes in the water capacity and oxygen availability of the fen soils.

Twenty-nine genes of JP12^T^ were identified as antibiotic resistance genes, suggesting a role in the survival of *Acidobacteriota* strains despite their low biomass production and a potential growth advantage against fast-growing bacteria of the *Pseudomonadota*, *Actinomycetota* and *Bacteroidota*. The genome of JP12^T^ was analysed via the antiSMASH pipeline (https://antismash.secondarymetabolites.org) [38]. The options ‘relaxed detection strictness’ and ‘all possible extra features’ detected ten secondary metabolite regions in strain JP12^T^, encoding either terpene (or terpene-precursor), hydrogen-cyanide RiPP-like (ribosomally synthesized and post-translationally modified peptides), T3PKS (type III polyketide synthases), NRPS-like (nonribosomal peptide synthetase) or T1PKS (type I polyketide synthases) compounds (Table S2). The presence of cbb3-type cytochrome-c-oxidase subunit I and putative bifunctional cbb3-type cytochrome-c-oxidase subunit II/cytochrome-c explains why strain JP12^T^ was able to survive under microaerophilic conditions in the fen soil, during the isolation process and under microaerophilic conditions in the laboratory. Genes for the degradation of complex carbon compounds, such as xylan (endo-1,4-β-xylanase, EC 3.2.1.8) and laminarin (laminarinase, EC 3.2.1.39), as detected in the genome of JP12^T^, confirm that strain JP12^T^ is adapted to the harsh conditions of the soil environment and is able to use complex hemicellulose as growth substrates. However, the ability to degrade complex substrates could not be proven under laboratory conditions. Interestingly, in the genome of strain JP12^T^, genes for gamma-hexachlorocyclohexane degradation, benzoate degradation via hydroxylation, bisphenol A degradation and fluorobenzoate degradation could be determined and could hint for a potential role of the strain in bio-restoration of contaminated soils.

## Morphological and physiological characterization

Strain JP12^T^ grew as pink, silk-mat-like, round colonies with 0.5–1.0 mm in diameter on SSE/HD 1:10 agar plates after 3 weeks of incubation at 28 °C. In liquid medium, JP12^T^ showed the formation of yellow-to-pale pink aggregates, which were too stable to be dissolved by normal shaking. However, during longer incubation periods in liquid culture, strain JP12^T^ seemed to lose this characteristic, and nearly no flocks were determined in the cultures anymore.

**Fig. 3.**
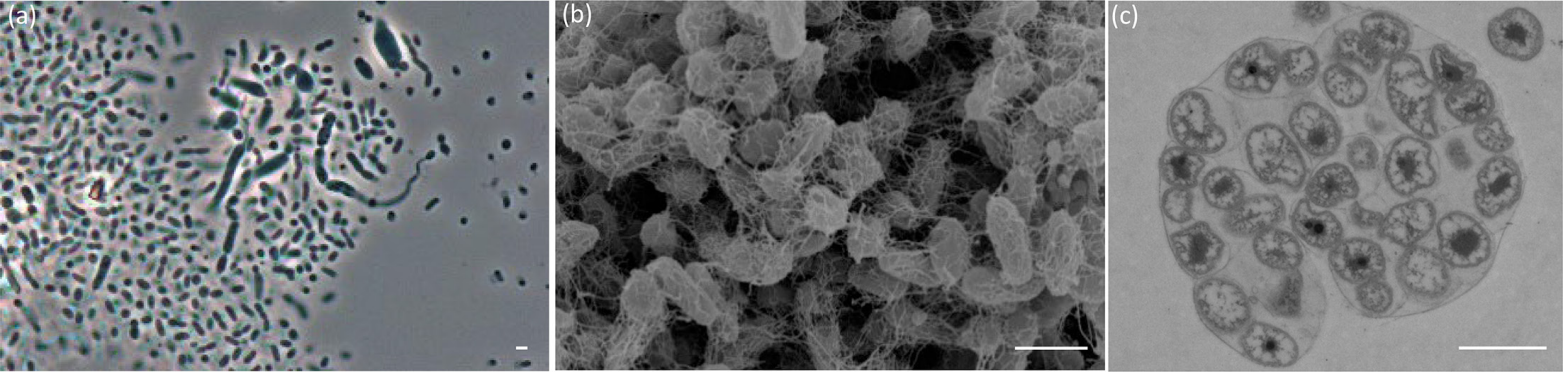
Phase-contrast photomicrograph (**a**), scanning electron micrograph (**b**) and transmission electron micrograph (**c**) of strain JP12^T^. Scale bars, 1 µm.

JP12^T^ was stained according to the Gram, India ink and malachite green methods, respectively, and subsequently examined by light microscopy (Zeiss Axio Scope A.1 equipped with an AxioCam MRc camera; Carl Zeiss, Jena, Germany) to determine cell wall structures, cell size, presence of capsules and endospores, as described before [39]. Strain JP12^T^ formed non-motile rods (0.45±0.10×1.20±0.35 µm in width and length, respectively; *n*=10 cells) that divided by binary fission (Fig. 3a). While a Gram-negative cell wall structure and the presence of capsules could be confirmed for JP12^T^, as for the two closely related *Occallatibacter* strains [10] and *Terracidiphilus gabretensis* S55^T^ [11], no endospores were detected, comparable to all other known *Acidobacteriota* strains. Additionally, the formation of aggregates, as seen in liquid cultures, could be confirmed by the presence of exopolysaccharide (EPS)-like structures in the scanning (Fig. 3b) and transmission electron micrographs (Fig. 3c).

Anaerobic growth of strain JP12^T^ tested negative in duplicates on SSE/HD 1:10 medium (pH 5.0) under anoxic conditions by the application of the Oxoid™ AnaeroGen™ Compact System for 6 weeks at 28 °C. Incubation of strain JP12^T^ for 4 weeks at 28 °C in a candle jar confirmed that strain JP12^T^ could grow under microaerophilic conditions and also showed a biomass production capacity comparable to that under oxic conditions. For better comparability with other strains of the *Occallatibacter*–*Terracidiphilus* clade, the physiological tests were performed under oxic conditions.

## Growth ranges

Liquid SSE/HD 1:10 medium was used to test the growth ranges and optima of JP12^T^ regarding temperature (4, 10, 15, 20, 22, 24, 27, 30, 36, 40 and 45 °C) and pH values (2.5–10). The pH of the medium for the temperature test was buffered at pH 5.0, whereas the pH of the medium for the pH tolerance test was buffered with the substrates MES (used for pH values 2.5–6.5), HEPES (used for pH values 7.0–8.0), HEPPS (used for pH value 8.5) and CHES (used for pH values 9.0–10.0). All buffers displayed a final concentration of 10 mM. NaCl concentrations of 0%, 0.25%, 0.50%, 1.0%, 3.0%, 5.0%, 7.5% and 10% (w/v) were adjusted in liquid HD 1:10 medium (DSMZ medium 1124; https://www.dsmz.de/microorganisms/medium/pdf/DSMZ_Medium1124.pdf) to determine the salinity tolerance. JP12^T^ showed growth at temperatures between 4 and 40 °C (best between 24 and 30 °C), grew at pH values between 3.7 and 6.0 and showed best growth at pH values of 4.1–5.6. JP12^T^ could only endure minor amounts of NaCl (0–0.5%, best at 0.25% NaCl, w/v).

## Nutritional tests and enzymatic activities

For the substrate utilization test of strain JP12^T^ (including different substrates such as sugars, keto acids, organic acids, amino acids, alcohols, casamino acids, laminarin, peptone, yeast extract, casein hydrolysate and Tween 80), triplicates of liquid 1x SSE solution [18] were adjusted at pH 5.0, supplemented with 1 ml l^−1^ vitamin solution, 1 ml l^−1^ trace element SL-10 solution and the respective test substrates at concentrations between 0.5 and 10 mM or rather 0.001% and 0.05% (w/v; Table S3). After 8 weeks of incubation in the dark at 20 °C, weak growth or rather growth was positively evaluated if the OD_660nm_ mean values (optical density) of the substrates exceeded the control values (culture without substrate) by a factor of 1.2–1.5 or by a factor larger than 1.5, respectively. JP12^T^ grew on various organic acids and amino acids (Table S4; further details are given in the species description). Interestingly, JP12^T^ grew well on DSMZ medium 1426 containing glucose, yeast extract and peptone, but did not grow on any of these substrates tested separately. Other acidobacterial strains, such as *Blastocatella fastidiosa* A2-16^T^, *Brevitalea aridisoli* Ac_11_E3^T^, *Arenimicrobium luteum* Ac_12_G8^T^ and *Stenotrophobacter terrae* Ac_28_D10^T^, also seemed to prefer a combination of substrates for growth (growth in DSMZ medium 1426) rather than the use of certain single substrate compounds [39].

After 4 weeks of incubation of strain JP12^T^ on solidified SSE medium supplemented with 1 ml l^−1^ vitamin solution, 1 ml l^−1^ trace element SL-10 solution, 0.005% (w/v) yeast extract and complex substrates such as starch, cellulose, pectin, colloidal and non-colloidal chitin, polygalacturonic acid and xylan (0.5 g l^−1^ final concentration), the degradation potential was detected by staining the agar with the respective staining solutions and observing the appearance of clearing zones as reported earlier [40]. No clearing zones, and therefore no degradation potential, could be confirmed for the different tested complex substrates. Additionally, API^®^ZYM and API^®^20NE galleries (bioMérieux, Marcy-l'Étoile, France) were employed to determine further exoenzyme activity potential, the formation of indole, aesculin degradation, urease activity, reduction of nitrogen compounds and the assimilation capacity of different carbon compounds (Table S5). Catalase and cytochrome-c-oxidase activities were tested by standard protocols [41,42]. JP12^T^ displayed only minor catalase activity reactions and no reaction of cytochrome-c-oxidase.

## Chemotaxonomic characterization

For fatty acids, polar lipids and respiratory quinone analyses, cells of JP12^T^, *Occallatibacter riparius* DSM 25168^T^/DSM 25169, *O. savannae* DSM 25170^T^ and *Terracidiphilus gabretensis* DSM 100509^T^ were grown in SSE/HD 1:10 medium (pH 5.5) at 25 °C for 14 days. Only *Telmatobacter bradus* DSM 23630^T^ was grown in half-strength R2A liquid medium (https://bacmedia.dsmz.de/medium/830) at 25 °C for 14 days, as the strain could not be grown in SSE/HD 1:10 medium at all.

To extract membrane-spanning lipids such as *iso*-diabolic acid, a method adapted from Sinninghe Damsté *et al*. [43] was employed. A total of ∼20 mg of wet biomass was treated with 1,900 µl methanol and 100 µl of 1M HCl solution in a sealed reaction tube and heated at 65 °C for 3 h. Following this, the pH value was adjusted to 7 using a KOH solution (2 M KOH:methanol, 1:1, v/v). The sample was then evaporated to dryness. Methylation was carried out by adding 1 ml methanol and 50 µl concentrated sulphuric acid, followed by overnight incubation at 50 °C. Subsequently, 500 µl of saturated NaCl solution was added, and then the mixture was extracted twice with 1 ml hexane:dichloromethane (4:1, v/v). The combined organic phases were evaporated and redissolved in methyl tert-butyl ether for analysis via GC with flame ionization detection (on an Agilent Technologies 6890B).

Equivalent chain length values were calculated analogously to the Sherlock Microbial Identification System to provide peak naming according to the TSBA6 database. Identification of fatty acids was performed via mass spectrometry [44]. The position of double bonds of monosaturated fatty acids was determined by derivatization with dimethyl sulphide [45].

The fatty acid profiles of JP12^T^ showed very similar composition patterns, with *iso*-C_15:0_ (26.5%), *iso*-C_17:1_ *ω*7*c* (11.3%), *iso*-C_17:0_ (6.3%) and *iso*-diabolic acid (49.2%) as the major fatty acids, compared with its closest phylogenetic relatives – *O. riparius* DSM 25168^T^/DSM 25169, *O. savannae* DSM 25170^T^, *T. gabretensis* DSM 100509^T^ and *T. bradus* DSM 23630^T^ (Table 2). Only the presence of an *iso*-diabolic acid likely isomer (16.9%) in *T. gabretensis* DSM 100509^T^ and a threefold higher amount of *iso*-C_15:1_ *ω*7*c* (3.0%) in *T. bradus* DSM 23630^T^ differed the strains within the *Occallatibacter*–*Telmatobacter*–*Terracidiphilus* clade from each other (Table 2).

**Table 2.** Fatty acids composition of strain JP12^T^ and its closest phylogenetic relatives Strains: 1, JP12^T^; 2, *Occallatibacter riparius* DSM 25168^T^; 3, *O. riparius* DSM 25169; 4, *O. savannae* DSM 25170^T^; 5, *Terracidiphilus gabretensis* DSM 100509^T^ and 6, *Telmatobacter bradus* DSM 23630^T^. The values are given in percentages and were obtained during the current study. Values larger than 5 are highlighted in bold. –, Not detected.

Fatty acid/strain	1	2	3	4	5
***iso*-C_13:0_**	–	–	–	–	–
***iso*-C_14:0_**	–	–	–	0.5	–
**C_14:0_**	0.3	–	0.2	0.1	–
***iso*-C_15:1_ *ω*7*c***	0.9	–	–	0.1	–
***iso*-C_15:1_ *ω*5*c***	0.1	0.1	0.2	0.2	–
***iso*-C_15:0_**	**26.5**	**33.9**	**40.2**	**29.8**	**20.7**
***anteiso*-C_15:0_**	0.3	0.6	0.2	0.2	0.2
**C_15:0_**	0.1	–	–	–	–
***iso*-C_16:0_**	0.5	0.2	0.2	1.3	0.2
**C_16:1_ *ω*10*c***	–	–	–	0.3	–
**C_16:1_ *ω*9*c***	–	–	–	–	–
**C_16:1_ *ω*7*c***	0.6	0.4	0.6	0.7	0.7
**C_16:0_**	2.2	1.1	1.4	1.4	3.2
***iso*-C_17:1_ *ω*7*c***	**11.3**	**18.8**	**20.3**	**21.2**	**15.4**
***anteiso*-C_17:1_ *ω*7*c***	0.2	0.7	0.7	0.6	0.4
***iso*-C_17:0_**	**6.3**	**6.6**	**7.2**	**9.0**	**10.9**
***anteiso*-C_17:0_**	0.9	0.5	1.2	0.7	0.6
**C_17:0_**	0.1	–	–	–	–
**C_18:1_ *ω*9*c***	–	–	0.3	–	0.1
**C_18:1_ *ω*7*c***	0.1	–	–	–	–
**C_18:0_**	0.3	0.4	0.4	0.5	0.8
***iso*-C_19:0_**	0.1	–	–	0.2	–
**Likely isomere of *iso*-diabolic acid**	–	–	–	–	**16.9**
***iso*-diabolic acid**	**49.2**	**36.7**	**26.8**	**33.2**	**29.8**

Respiratory quinones were extracted from 5 mg of wet biomass via solid-phase extraction and analysed via reversed-phase HPLC coupled to a diode array detector [44]. MK-8 was detected as the sole respiratory quinone of JP12^T^.

Polar lipids of strain JP12^T^ were extracted using a modified Bligh and Dyer extraction [46] and analysed via HPLC-MS as described previously [47]. Phosphatidylglycerol, phosphatidylethanolamine and ornithine-containing lipids were identified as major polar lipids. In addition, lysophosphatidylethanolamine, phosphatidylinositol, diphosphatidylglycerol and high-mass intact polar lipids occurred in small amounts.

## Taxonomic conclusion

Although the 16S rRNA phylogenetic trees identified *Telmatobacter bradus* TPB6017^T^ as the phylogenetically closest relative of the strain JP12^T^, the results of the fatty acid analysis, phylogenomic tree and genome comparison results (POCP and AAI values) suggest that JP12^T^ would represent a new species within the *Occallatibacter* genus. In addition, JP12^T^ and *T. bradus* TPB6017^T^ differed in physiological traits: *T. bradus* TPB6017^T^ does not form any aggregates or EPS-like structures and is a facultatively anaerobic, chemo-organotrophic organism, unlike JP12^T^. While all *Occallatibacter* strains and *Terracidiphilus gabretensis* DSM 100509^T^ were able to grow on SSE/HD 1:10, *T. bradus* DSM 26360^T^ was not able to grow on this medium at all.

Moreover, the genome comparison results, i.e. AAI and POCP values, and the phylogenomic tree analysis of the *Occallatibacter*–*Telmatobacter*–*Terracidiphilus* clade underlined our findings that *T. gabretensis* S55^T^ would represent a species within the *Occallatibacter* genus and that *T. bradus* TPB6017^T^ should not be merged with the *Occallatibacter* genus.

Therefore, based on the morphological, physiological and phylogenetic characteristics and whole-genome similarity indices, we propose JP12^T^ as a new species within the acidobacterial genus *Occallatibacter*–*Occallatibacter bavaricus* sp. nov. (Table 3) and the reclassification of *Terracidiphilus gabretensis* as *Occallatibacter gabretensis* comb. nov.

**Table 3.** Characteristics of JP12^T^ compared with the phylogenetically closest related type strains Strains: 1, JP12^T^; 2, *Occallatibacter riparius* 277^T^ [10]; 3, *O. savannae* A2-1c^T^ [10]; 4, *Terracidiphilus gabretensis* S55^T^ [11] and 5, *Telmatobacter bradus* TPB6017^T^ [5]. Major lipids are highlighted in bold.

Characteristic	1	2	3	4	5
Source	Fen soil	River bank soil	Savannah soil	Coniferous soil	Peatland soil
Cell shape	Rod	Rod	Rod	Rod	Rod
Cell length/width (µm)	1.2×0.45	1.0–2.0 0.6–0.7	1.0–2.5 0.5–0.7	0.6–1.2 0.5–0.8	2–10 0.4–0.6
Cell division	BF	BF	BF	nd	BF
Motility	−	(+)	(+)	+	+
Pigmentation	Yellow-to-pale pink	Cream pink	Bright pink	White	White beige
Spores	−	−	−	−	−
Capsule/EPS formation	+	+	+	+	−
Oxidase	−	−	−	−	−
Catalase	(+)	+	+	−	(+)
Quinone	MK-8	MK-8	MK-8, MK-7	MK-8	MK-8
Metabolism	AC	AC	AC	AC	AnC
NaCl tolerance (%, w/v)	0–0.5	0–1.0	0–0.5	0	0–0.1
Temperature range/optimum (°C)	4-40 24-30	11-40 26-37	11-40 29–40	12-30 20-24	4-35 20-28
pH range/optimum	3.7–6.0 4.1–5.6	3.5–8.5 5.0–6.5	3.5–6.5 4.0–5.5	3–6 4–5	3.0–7.5 4.5–5.0
DNA G+C content (mol%)	56.4	59.6	58.5	57.3	57.6
Lipids	**PG, PE, OL,** LPE, PI, DPG, IPLs	**PE, LPE, OL,** PG, DPG, IPLs	**PG, PE, PI, OL,** DPG, IPLs	**PG, PE, OL,** IPLs	**PG, PE, LPE, PC, OL,** LPC, IPLs

+, Positive; −, negative; (+), weak reaction detected; nd, no data available.

The data were obtained from the corresponding literature unless otherwise noted.

AC, aerobic chemoheterotrophic; AnC, anaerobic chemoheterotrophic; BF, binary fission; DPG, diphosphatidylglycero; GPL, glycorophospholipid; IPL, intact polar lipid; LL, lysine lipid; LPC, lysophosphatidylcholine; LPE, lysophophatidylethanolamine; OL, ornithine lipid; PC, phosphatidylcholine; PE, phosphatidylethanolamin; PG, phosphatidylglycerol; PL, phosphatidylinositol.

## Description of *Occallatibacter bavaricus* sp. nov.

*Occallatibacter bavaricus* (ba.va’ri.cus. N.L. masc. adj. bavaricus, pertaining to Bavaria, to indicate the original habitat of the type strain of the species, which was isolated from fen soil close to Bayreuth, Germany).

Cells are Gram-negative, non-motile rods with 0.45 µm in width and 1.20 µm in length and divide by binary fission. Cytochrome-c-oxidase activity is negative, and catalase activity is weakly positive. Neither motility nor spores are observed, but EPS-like structures are present. Capsules are present. In liquid cultures, strong yellow-to-pale pink aggregates are formed, which cannot be dissolved by shaking. Long-term incubations and several transfers to fresh media reduce this effect. On agar plates, pink, silk-mat-like, round colonies with 0.5–1.0 mm in diameter are formed.

Grows occurs at temperature values of 4–40 °C (best between 24 and 30 °C) and at pH values of 3.7–6.0 (best between 4.1 and 5.6). Small amounts of NaCl (0–0.5%; best 0.25% NaCl, w/v) support the growth.

The major fatty acids are *iso*-C_15:0_, C_16:0_*, iso*-C_17:1_ *ω*7*c*, *iso*-C_17:0_ and *iso*-diabolic acid. MK-8 is the major respiratory quinone. Phosphatidylglycerol, phosphatidylethanolamine and ornithine-containing lipids are the major polar lipids. In addition, glycophospholipid, lysine lipid and high-mass intact polar lipid occur in small amounts. Utilizes arabinose, *N*-acetylgalactosamine, maleic acid, 1,2-butandiole, 2,3-butandiole, 1,2-propandiole, alanine, arginine, asparagine, isoleucine, ornithine, proline, acetate, butyrate, β-hydroxybutyrate, γ-hydroxybutyrate, isobutyrate, tyrosine, serine, phenylalanine, glycine, leucine, histidine, valine, methionine, threonine, succinate, nicotinic acid, Tween 80, adipate, protocatechuate, shikimate, aspartate, glutamate, laminarin, malate, citrate, tartrate, fermented rumen extract, isovaleric acid, heptanoic acid, fumarate, trimethoxybenzoate, crotonate, 2-oxoglutarate, sodium pyruvate, acetoin, ascorbate, glyoxylate, 2-oxovalerate and 2-oxogluconate. Grows weakly on caproate, caprylate, dulcitol, ethylene glycol, erythrose, erythrulose, α-hydroxybutyrate, isocitrate, levulinate, 2-oxogluconate, cysteine, glutamine, benzoate, tryptophan and formate. No growth is observed on glucose, lactose, fructose, cellobiose, galactose, mannose, melezitose, raffinose, fucose, sorbose, lyxose, maltose, rhamnose, sucrose, trehalose, xylose, adonitol, arabitol, mannitol, myo-inositol, sorbitol, xylitol, l-lysin-HCl, hydroxy-l-proline, casamino acids, casein hydrolysate, yeast extract, glycolate, malonate, propionate, oxaloacetate, lactate, butanol, ethanol, glycerin, methanol, propanol, peptone, *N*-acetylglucosamine, glucosamine, gluconate, glucuronate, lyxitol, crotonate, 2-oxoglutarate, sodium pyruvate, acetoin, ascorbate or glyoxylate.

Positive for leucine arylamidase, acid phosphatase, naphthol-AS-BI-phosphohydrolase, β-galactosidase (API^®^ZYM and API^®^20NE), β-glucuronidase, α-glucosidase, β-glucosidase (API^®^ZYM and API^®^20NE), *N*-acetylglucosaminidase and α-fucosidase. Weakly positive for esterase C4, esterase lipase C8, valine arylamidase and α-galactosidase.

The type strain is JP12^T^ (=DSM 110680^T^=CECT 30267^T^) and was isolated from fen soil collected at Schlöppnerbrunnen II in Germany. The DNA G+C content of the type strain is 56.4 mol%, and the GenBank/EMBL/DDBJ accession numbers for the 16S rRNA gene sequence and the genome sequence are OQ656429 and CP121196, respectively.

## Description of *Occallatibacter gabretensis* comb. nov.

*Occallatibacter gabretensis* (ga.bret.en’sis. N.L. masc. adj. *gabretensis*, pertaining to Gabreta, the Celtic name of the Bohemian Forest, the mountain range in central Europe where the type strain was isolated).

Basonym: *Terracidiphilus gabretensis* Garcia-Fraile *et al*. 2016 [11].

The description of the species is provided by García-Fraile *et al*. [11]. According to phylogenetic and phylogenomic data, *Terracidiphilus gabretensis* was transferred into *Occallatibacter*.

The type strain of the species is S55^T^ (=CECT 8791^T^=NBRC 111238^T^).

The DNA G+C content of the type strain is 57.3 mol%, and the GenBank/EMBL/DDBJ accession numbers for the 16S rRNA gene sequence and the genome sequence are KP120762 and LAIJ01000000, respectively.

## Emended description of the genus *Occallatibacter* Foesel *et al.* 2016

The description is as given by Foesel *et al*., 2016 [10], with the following modifications: mostly catalase-positive but oxidase-negative. Tumbling events as motility and polysaccharide degradation might occur. The DNA G+C content ranges from 56.4 to 59.6 mol%.

## Supplementary material

10.1099/ijsem.0.007086 Uncited Supplementary Material 1.

## Abbreviations

AAI: average amino acid identity
ANI: average nucleotide identity
BV-BRC: Bacterial and Viral Bioinformatics Resource Center
dDDH: digital DNA–DNA hybridization
DSMZ: Leibniz Institute DSMZ - German Collection of Microorganisms and Cell Cultures GmbH
EPS: exopolysaccharide
HD: Hefe - Dextrose (Yeast - Glucose)
NRPS: nonribosomal peptide synthetase
POCPs: percentage of conserved proteins
RiPP: ribosomally synthesized and post-translationally modified peptides
SSE: soil solution equivalent
T1PKS: type I polyketide synthases
T3PKS: type III polyketide synthases
