# Supplementary material for: Occallatibacter bavaricus sp. nov., a new representative of the Acidobacteriota isolated from fen soils, reclassification of Terracidiphilus gabretensis as Occallatibacter gabretensis comb. nov. and emended description of the genus Occallatibacter
Source: Int J Syst Evol Microbiol. 2026 Feb 25;76(2):007086. doi: 10.1099/ijsem.0.007086 (PMC12935467; doi:10.1099/ijsem.0.007086)
Supplement: Uncited Supplementary Material 1. [file ijsem-76-07086-s001.pdf]

Supplementary Material

Occallatibacter bavaricus* sp. nov. – a new representative of the *Acidobacteriota* isolated from fen soils, reclassification of *Terracidiphilus gabretensis* as *Occallatibacter gabretensis* comb. nov. and emended description of the genus *Occallatibacter

Katharina J. Huber[1†]*, János Papendorf[1†], Carolin Pilke[1], Petra Büsing[1], Boyke Bunk[2], Cathrin Spröer[2], Sarah Kirstein[3], Jacqueline Wolf[3], Meina Neumann-Schaal[3,4], Manfred Rohde[5], Michael Pester[1,6]

[1] Department of Microorganisms, Leibniz Institute DSMZ – German Collection of Microorganisms and Cell Cultures, Braunschweig, Germany

[2] Bioinformatic Services, Leibniz Institute DSMZ – German Collection of Microorganisms and Cell Cultures, Braunschweig, Germany

[3] Department of Metabolomics & Services, Leibniz Institute DSMZ – German Collection of Microorganisms and Cell Cultures, Braunschweig, Germany

[4] Braunschweig Integrated Centre of Systems Biology (BRICS), Braunschweig, Germany

[5] Department of Medical Microbiology, Central Facility for Microscopy, Helmholtz Centre for Infection Research, Braunschweig, Germany

[6] Institute of Microbiology, Technische Universität Braunschweig, Braunschweig, Germany

Keywords: Acidobacteriota, soil bacteria, fen soils

Repositories: The GenBank/EMBL/DDBL accession numbers for the 16S rRNA and genome sequence of the strain JP12^T are OQ656429 and CP121196, respectively.

Data summary:

All supporting data have been provided within the article or through supplementary data files.

[†] Shared first authorship.

* Correspondence: K. Huber, Leibniz-Institute DSMZ – German Collection of Microorganisms and Cell Cultures GmbH, Inhoffenstraße 7B, 38124 Braunschweig, Germany.

Tel.: +49-0531-2616-365; Fax: +49-531-2616-415; Email: Katharina.Huber@dsmz.de

Material and Methods –Colony PCR

26 turbid wells of liquid medium approach and 42 colonies on the agar plates were screened for the presence of *Acidobacteriota* by colony-PCR. Either 1 µl of the resuspended grown well or a pipet tip of biomass resuspended in PCR water were used for PCR.

The respective PCR mixture included 39.75 µl PCR-water, 5 µl Dream Taq Buffer (10x), 1 µl dNTPs (10 mM each), 2 µl forward primer [27f (10 µM)], 2 µl reverse primer [1492r (10 µM)], 0.25 µl DreamTaq Green-Polymerase (5 U/µl; Thermo Scientific) and 1.0 µl DNA.

The DNA was subsequently amplified by the colony PCR programme including an initial denaturation step at 95°C for 5 min and 35 cycles of 95°C for 30 s, 52°C for 30 s and 72°C for 90 s followed. The PCR reaction was finalized by an elongation step at 72°C for 7 min and a cooldown phase at 4°C for eternity.

Material and Methods – Transmission electron microscopy

For transmission electron microscopy (TEM) samples were fixed with 2% glutaraldehyde in culture medium, left for 30 min on ice, then further fixed with 5% formaldehyde and left for 5 h at 7°C. Then the samples were washed twice with 0.1 M HEPES buffer and immobilized with 2% water agar. Dehydration was achieved with a graded series of ethanol (10%, 30%, 50%) for 30 min on ice. The 70% ethanol step containing 2% uranyl acetate was performed overnight at 7°C, followed by the 90% ethanol step for 30 min on ice. The 100% ethanol step was performed twice for 30 min at room temperature. For embedding LRWhite resin (hard formular) was used. Polymerization of the LRWhite was carried out at 50°C for 2 days. Ultrathin sections were cut with a diamond knife and collected with a butvar coated copper grid. Post-staining of sections was done with 2% aqueous uranyl acetate for 3 min. After washing in distilled water and air-drying samples were examined in an EM 910 transmission electron microscope (Zeiss, Oberkochen, Germany) at an acceleration voltage of 80 kV. Images were taken at calibrated magnifications using a line replica and recorded digitally with a Slow-Scan CCD-Camera (ProScan, 1024x1024, Scheuring, Germany) applying the ITEM-Software (Olympus Soft Imaging Solutions, Münster, Germany).

Material and Methods – Field emission scanning electron microscopy

For field emission scanning electron microscopy (FESEM) HEPES buffer washed samples as described above were adsorbed onto poly-L-lysine coated cover slips (12 mm in diameter) for 10 min, fixed with 1% glutaraldehyde in TE buffer and washed twice with HEPES buffer. Samples were then dehydrated with a graded series of acetone (10, 30, 50, 70,90, 100%) for 10 min each step on ice. The 100% acetone step was repeated at room temperature before samples were critical point dried with liquid CO_2 (Leica, CPD 300) and sputter coated with gold-palladium (Bal-Tec, SCD 500). Samples were examined in a Zeiss Merlin field emission scanning electron microscope at an acceleration voltage of 5 kV using the SE

(secondary electron)-lens and Everhart-Thornley SE-detector in a 75:25 ratio. Images were recorded applying the SmartSEM software version 6.06.

Material and Methods – Genome sequencing

For the genome sequencing of the strain JP12^T genomic DNA extraction was carried out on MasterPure™ Gram Positive DNA Purification Kits from Epicentre®Biotechnologies Germany according to the manufacturer´s instructions. SMRTbell™ template libraries were prepared according to the instructions from PacificBiosciences, Menlo Park, CA, USA, following the Procedure & Checklist – Preparing Multiplexed Microbial Libraries Using SMRTbell® Express Template Prep Kit 2.0. Briefly, for preparation of 10kb libraries 1 µg genomic DNA was sheared using g-tubes™ from Covaris, Woburn, MA, USA according to the manufacturer´s instructions. DNA was end-repaired and ligated to barcoded adapters applying components from the SMRTbell Express Template Prep Kit 2.0 from Pacific Biosciences, Menlo Park, CA, USA. Reactions were carried out according to the manufacturer´s instructions. Samples were pooled according to the calculations provided by the Microbial Multiplexing Calculator. Conditions for annealing of sequencing primers and binding of polymerase to purified SMRTbell™ template were assessed with the Calculator in SMRT®link, PacificBiosciences, Menlo Park, CA, USA. Libraries were sequenced on the Sequel*II* (PacificBiosciences, Menlo Park, CA, USA) taking one 15 h movie per SMRT cell. Long read genome assembly was performed with the "Microbial Assembly" protocol included in SMRTlink version 10.2 using default parameters with exception of the target genome size, which was set to 5 Mbp. One circular chromosomal contig of 6 Mbp was obtained and adjusted to *dnaA*. Identification replication genes has been done based on BLAST, circularization and rotation to the replication genes has been performed by genomecirculator.jar tool (https://github.com/boykebunk/genomefinish). Subsequent genome annotation was based on Prokka 1.8 (1) with subsequent manual curation for the strain JP12^T.

Supplementary Table 1. Summary of genomic features of the strain JP12^{T}.

	**JP12^{T}**
**Genome length [bp]**	5,987,865
**G + C content [mol%]**	56.4
**sequencing coverage values**	487
**CDS regions**	5040
**rRNA**	3
**tRNA**	76

Supplementary Table 2. Overview of antiSMASH analysis results of the strain JP12^{T}.

Region	Type
1	terpene-precursor
2	hydrogen-cyanide
3	RiPP-like
4	terpene
5	terpene
6	RiPP-like
7	NRPS-like
8	T1PKS
9	T3PKS
10	terpene-precursor

Supplementary Table 3. Single substrate concentration used for determination of substrate range of the strain JP12^T in liquid culture and on agar plates*.

Substrate	Concentration [mM]
Arabinose	5
Cellobiose	5
Erythrose	5
Erythrulose	5
Fructose	5
Fucose	5
Galactose	5
Glucose	5
Lactose	5
Lyxose	5
Maltose	5
Mannose	5
Melezitose	5
Raffinose	5
Rhamnose	5
Sorbose	5
Sucrose	5
Trehalose	5
Xylose	5
Glucosamine	5
N-acetylglucosamine	5
N-acetylgalactosamine	5
Acetoin	5
Adonitol	5
Arabitol	10
Dulcitol	5
Lyxitol	5
Mannitol	5
Myo-Inositol	5
Sorbitol	5
Xylitol	5
Alanine	5
Arginine	5
Asparagine	2
Aspartate	2
Cysteine	2
Glutamate	2
Glutamine	2
Glycine	5

Histidine	5
Hydroxy-Proline	5
Isoleucine	2
Leucine	5
Lysine	5
Methionine	5
Ornithine	2
Phenylalanine	5
Proline	2
Serine	2
Threonine	5
Tryptophan	1.25
Tyrosine	5
Valine	5
Adipate	5
Acetate	5
Ascorbate	5
Benzoate	5
Trimethoxybenzoate	5
Butyrate	2.5
α-Hydroxybutyrate	2.5
β-Hydroxybutyrate	2.5
γ-Hydroxybutyrate	2.5
Isobutyrate	2.5
Caproate	5
Caprylate	5
Citrate	2
Isocitrate	5
Crotonate	5
Formate	2.5
Fumarate	5
Gluconate	5
2-Oxogluconate	5
Glucuronate	5
2-Oxoglutarate	5
Glycolate	5
Glyoxylate	5
Heptanoic acid	5
Isovalerate	0.5
Laevulinate	5
Lactate	2
Malate	5

Maleic acid	5
Malonate	5
Nicotinic acid	2
Oxaloacetate	5
Propionate	5
Protocatechuate	5
Pyruvate	10
Shikimate	5
Succinate	10
Tartrate	2
2-Oxovalerate	5
Butanol	5
1,2-Butandiol	5
2,3-Butandiol	5
Ethanol	5
Ethylene glycol	5
Glycerol	5
Methanol	2
Propanol	5
1,2-Propandiol	5
Fermented rumen extract	5
**Substrate**	**Concentration [% w/v]**
Laminarin	0.05
Tween 80	0.001
Casamino acids	0.05
Casein hydrolysate	0.05
Peptone	0.05
**Substrate***	**Concentration [mg l^{-1}]**
Starch	500
Cellulose	500
Xylan	500
Polygalacturonic acid	500
Chitin	500
Pectin	500

Supplementary Table 4: Substrate spectrum of JP12^T compared with the phylogenetically next related type strains.
Strains: 1, JP12^T; 2, *Occallatibacter riparius* 277^T (2); 3, *Occallatibacter savannae* A2-1c^T (2); 4, *Terracidiphilus gabretensis* S55^T (3); 5, *Telmatobacter bradus* TPB6017^T (4).
+, positive; -, negative; (+), weak growth detected; ND, no data available.

Carbon sources utilized	1	2	3	4	5
Arabinose	+	-	-	+	+
Cellobiose	-	+	+	+	+
Erythrose	(+)	ND	ND	ND	-
Erythrulose	(+)	ND	ND	ND	-
Fructose	-	+	+	+	+
Fucose	-	(+)	+	+	-
Galactose	-	+	+	+	+
Glucose	-	+	+	+	+
Lactose	-	+	+	ND	+
Lyxose	-	-	-	ND	ND
Maltose	-	+	+	+	+
Mannose	-	+	+	+	+
Melezitose	-	+	+	+	+
Raffinose	-	+	+	+	+
Rhamnose	-	+	+	ND	+
Sorbose	-	-	-	+	-
Sucrose	-	+	+	+	+
Trehalose	-	+	+	+	-
Xylose	-	+	+	+	+
Glucosamine	-	ND	ND	+	ND
N-acetyl-glucosamine	-	ND	ND	+	-
N-acetyl-galactosamine	+	ND	ND	ND	ND
Acetoin	-	ND	ND	ND	ND
Adonitol	-	-	-	ND	-
Arabitol	-	-	-	ND	-
Dulcitol	(+)	ND	ND	ND	-
Lyxitol	-	ND	ND	ND	ND
Mannitol	-	-	-	+	-
Myo-Inositol	-	-	-	ND	-
Sorbitol	-	(+)	-	+	-
Xylitol	-	-	+	ND	ND
Alanine	+	-	+	+	ND
Arginine	+	(+)	-	+	-
Asparagine	+	ND	ND	ND	-
Aspartate	+	+	-	ND	ND
Cysteine	(+)	-	-	ND	-
Glutamate	+	+	-	-	ND
Glutamine	(+)	ND	ND	ND	-
Glycine	+	-	-	ND	-
Histidine	+	-	-	+	ND
Hydroxy-Proline	-	-	-	ND	ND
Isoleucine	+	-	-	ND	ND

Leucine	+	-	-	ND	ND
Lysine	-	+	-	ND	ND
Methionine	+	-	-	ND	-
Ornithine	+	+	+	ND	ND
Phenylalanine	+	-	+	ND	ND
Proline	+	-	+	ND	-
Serine	+	-	-	ND	ND
Threonine	+	-	-	+	-
Tryptophan	(+)	-	-	ND	-
Tyrosine	+	+	+	ND	-
Valine	+	-	-	ND	-
Adipate	+	ND	ND	ND	ND
Acetate	+	(+)	-	ND	-
Ascorbate	-	ND	ND	ND	ND
Benzoate	(+)	ND	ND	ND	ND
Trimethoxybenzoate	+	ND	ND	ND	ND
Butyrate	+	-	-	ND	-
α-Hydroxy-butyrate	(+)	ND	ND	ND	ND
β-Hydroxy-butyrate	+	ND	ND	+	ND
γ-Hydroxy-butyrate	+	ND	ND	ND	ND
Isobutyrate	+	ND	ND	ND	ND
Caproate	(+)	ND	ND	ND	-
Caprylate	(+)	ND	ND	ND	ND
Citrate	+	-	-	ND	-
Isocitrate	(+)	ND	ND	ND	ND
Crotonate	-	-	-	ND	ND
Formate	(+)	-	-	ND	-
Fumarate	+	-	-	+	-
Gluconate	-	+	+	+	ND
2-Oxogluconate	(+)/+	ND	ND	ND	ND
Glucuronate	-	ND	ND	ND	+
2-Oxoglutarate	-/+	ND	ND	ND	ND
Glycolate	-	-	-	ND	ND
Glyoxylate	-	ND	ND	ND	ND
Heptanoic acid	+	ND	ND	ND	ND
Isovaleric acid	+	-	-	ND	ND
Levulinate	(+)	ND	ND	ND	ND
Lactate	-	-	-	ND	-
Malate	+	-	-	ND	-
Maleic acid	+	ND	ND	+	ND
Malonate	-	-	-	ND	ND
Nicotinic acid	+	-	-	ND	ND
Oxaloacetate	-	-	-	ND	ND
Propionate	-	-	-	ND	-
Protocatechuate	+	ND	ND	ND	ND
Pyruvate	-	-	+	ND	+
Shikimate	+	ND	ND	ND	ND
Succinate	+	+	+	+	-
Tartrate	+	-	-	+	ND
2-Oxovalerate	(+)/+	ND	ND	ND	-

Butanol	-	-	-	ND	ND
1,2-Butandiol	+	ND	ND	ND	ND
2,3-Butandiol	+	ND	ND	ND	ND
Ethanol	-	-	-	+	-
Ethylene glycol	(+)	ND	ND	ND	ND
Glycerol	-	+	+	+	ND
Methanol	-	-	-	+	-
Propanol	-	-	-	ND	ND
1,2-Propandiol	+	ND	ND	ND	ND
Fermented rumen extract	+	ND	ND	ND	ND
Tween 80	+	-	-	ND	ND
Casamino acids	-	+	+	+	+
Casein hydrolysate	-	+	+	ND	ND
Peptone	-	+	+	+	+
Yeast extract	-	+	+	+	+
Laminarin	+	(+)	+	ND	+
Chitin	-	-	+	+	-
Cellulose	-	-	-	+	+
Pectin	-	-	+	ND	+
Starch	-	+	-	+	+
Xylan	-	-	-	+	+

The data was obtained from the corresponding literature unless otherwise noted.

Supplementary Table 5: Api®ZYM and API®20NE test spectrum of JP12[T] compared with the phylogenetically next related type strains.
Strains: 1, JP12[T]; 2, *Occallatibacter riparius* 277[T] (2); 3, *Occallatibacter savannae* A2-1c[T] (2); 4, *Terracidiphilus gabretensis* DSM 100509[T]; 5, *Telmatobacter bradus* TPB6017[T] (4).
+, positive; -, negative; (+), weak reaction detected; ND, no data available.

Characteristics	1	2	3	4[a]	5
Alkaline phosphatase	-	+	+	+	-
Esterase C4	(+)	+	(+)	-	+
Esterase lipase C8	(+)	+	(+)	-	-
Lipase C14	-	(+)	-	-	-
Leucine arylamidase	+	+	+	+	-
Valine arylamidase	(+)	+	(+)	(+)	+
Cysteine arylamidase	-	(+)	(+)	-	-
Trypsin	-	(+)	(+)	(+)	-
α-Chymotrypsin	-	(+)	(+)	-	-
Acid phosphatase	+	+	+	+	+
Naphtol-AS-BI-phosphohydrolase	+	+	(+)	-	+
α-Galactosidase	(+)	+	+	-	+
β-Galactosidase	+	+	+	-	+
β-Glucuronidase	+	+	(+)	-	+
α-Glucosidase	+	(+)	+	-	-[a]
β-Glucosidase	+	+	+	-	+
N-Acetylglucosaminidase	+	+	-	(+)	+
α-Mannosidase	-	-	-	-	-
α-Fucosidase	+	+	+	-	+
Nitrate reduction	-	-	-	-	-
β-Glucosidase	+	ND	ND	+	-
β-Galactosidase	+	ND	ND	+	+[a]
Urease	-	-	-	-	-

The data was obtained from the corresponding literature unless otherwise noted.

[a] Data obtained in the present study.

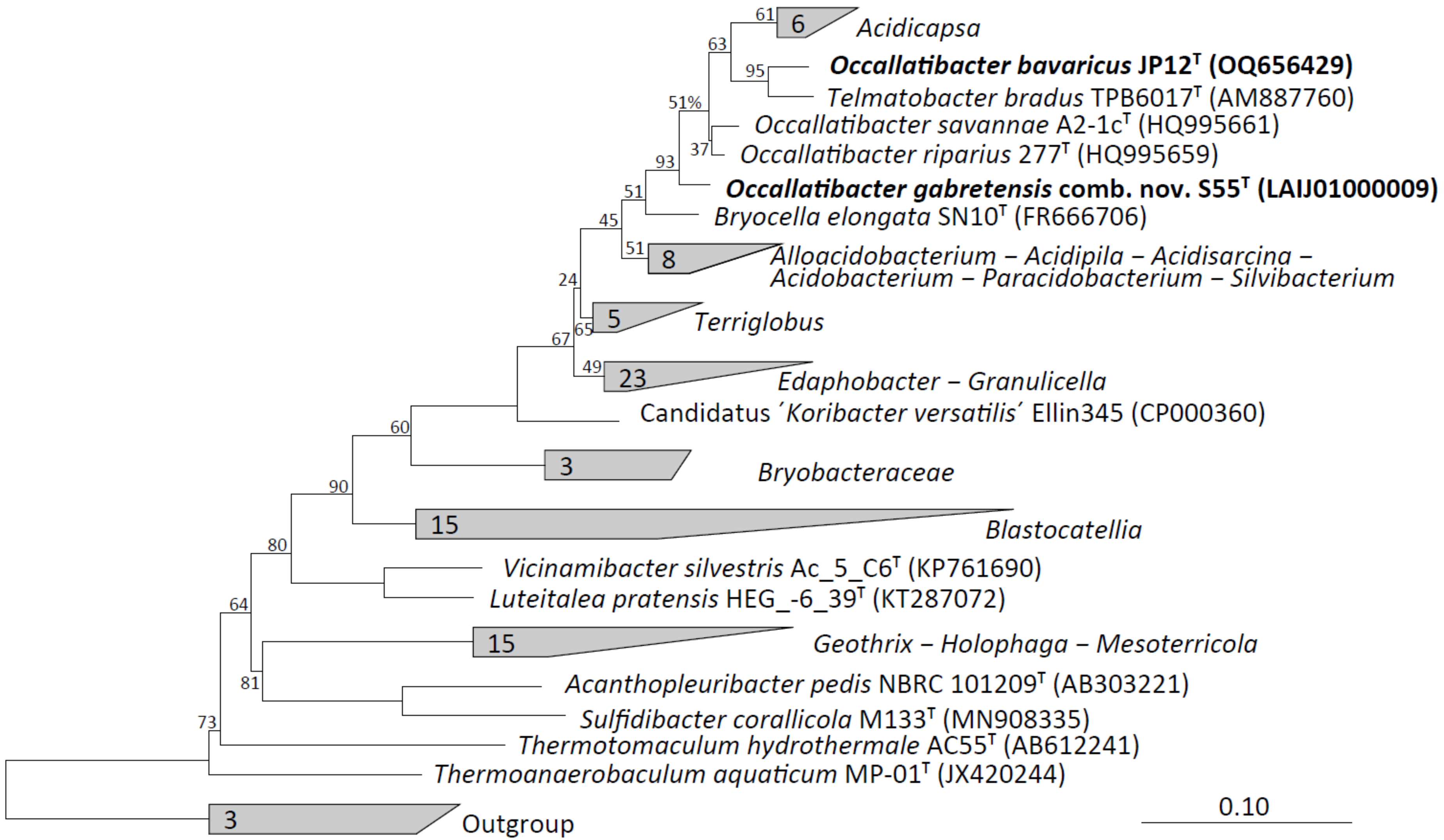

Supplementary Figure 1

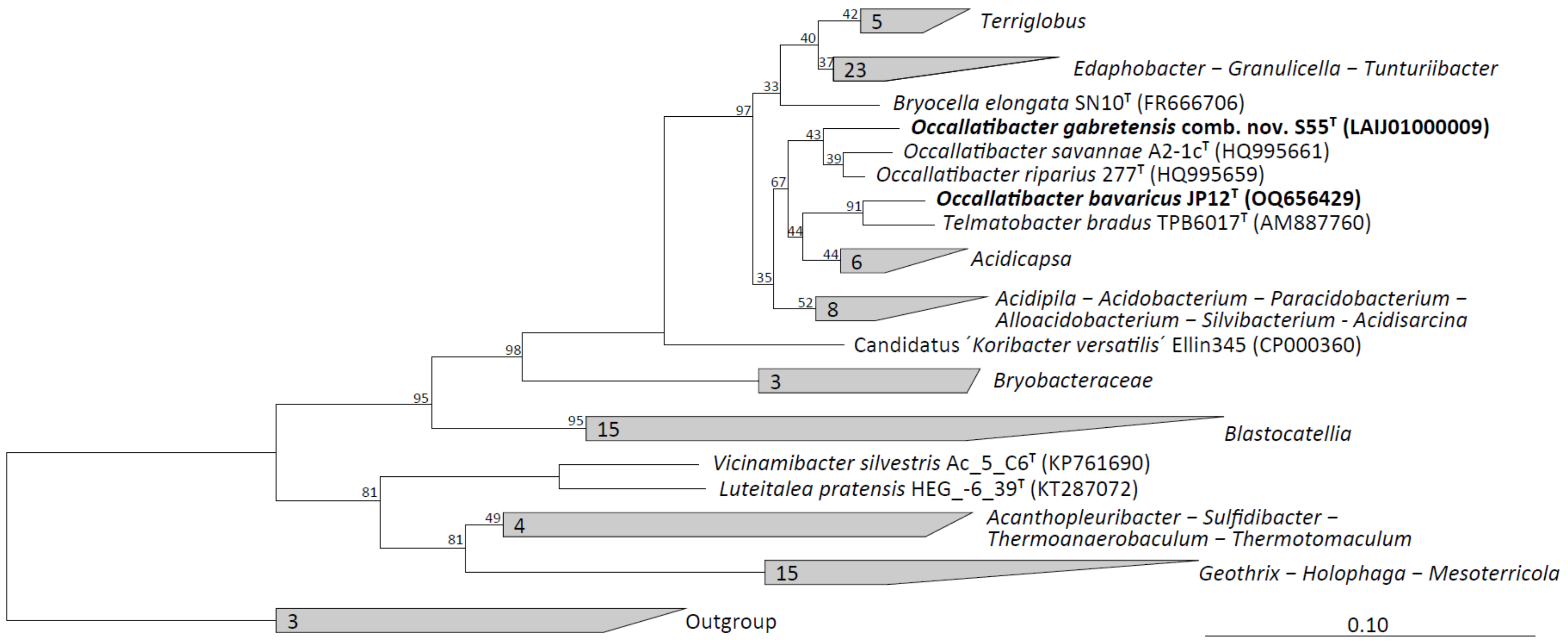

Supplementary Figure 2

FIGURE LEGENDS

Supplementary Figure 1. Maximum Likelihood phylogenetic Tree based on almost full-length 16S rRNA gene sequences showing the relationship of the strain JP12^T and related type strains. Bootstrap values are expressed as a percentages of 1000 replicates and are indicated at the respective branching points. The following sequences were used as outgroup: *Novipirellula rosea* LHWP3^T (JF748734), *Blastopirellula marina* DSM 3645^T (X62912) and *Pirellula staleyi* DSM 6068^T (CP001848). Bar indicates 10% nucleotide divergence.

Supplementary Figure 2. Maximum-Parsimony phylogenetic tree based on almost full-length 16S rRNA gene sequences showing the relationship of the strain JP12^T and related type strains. Bootstrap values are expressed as a percentages of 1000 replicates and are indicated at the respective branching points. The following sequences were used as outgroup: *Novipirellula rosea* LHWP3^T (JF748734), *Blastopirellula marina* DSM 3645^T (X62912) and *Pirellula staleyi* DSM 6068^T (CP001848). Bar indicates 10% nucleotide divergence.

REFERENCES

1. **Seemann T.** Prokka: rapid prokaryotic genome annotation. *Bioinformatics* 2014; 30:2068-2069
2. **Foesel BU, Mayer S, Luckner M, Wanner G, Rohde M *et al.*** *Occallatibacter riparius* gen. nov., sp. nov. and *Occallatibacter savannae* sp. nov., acidobacteria isolated from Namibian soils, and emended description of the family *Acidobacteriaceae*. *Int J Syst Evol Microbiol* 2016; 66:219-229
3. **García-Fraile P, Benada O, Cajthaml T, Baldrian P, Lladó S.** *Terracidiphilus gabretensis* gen. nov., sp. nov., an abundant and active forest soil acidobacterium important in organic matter transformation. *Appl Environ Microbiol* 2016; 82:560-569
4. **Pankratov TA, Kirsanova LA, Kaparullina EN, Kevbrin VV, Dedysh SN.** *Telmatobacter bradus* gen. nov., sp. nov., a cellulolytic facultative anaerobe from subdivision 1 of the Acidobacteria, and emended description of *Acidobacterium capsulatum* Kishimoto *et al.* 1991. *Int J Syst Evol Microbiol* 2012; 62:430-437
